# Oxytocin/Osteocalcin/IL-6 and NGF/BDNF mRNA Levels in Response to Cold Stress Challenge in Mice: Possible Oxytonic Brain-Bone-Muscle-Interaction

**DOI:** 10.3389/fphys.2019.01437

**Published:** 2019-11-27

**Authors:** Claudia Camerino, Elena Conte, Maria Rosaria Carratù, Adriano Fonzino, Marcello Diego Lograno, Domenico Tricarico

**Affiliations:** ^1^Department of Biomedical Sciences and Human Oncology (Section of Pharmacology), School of Medicine, University of Bari Aldo Moro, Bari, Italy; ^2^Department of Pharmacy–Pharmaceutical Sciences, University of Bari Aldo Moro, Bari, Italy

**Keywords:** osteocalcin, oxytocin, muscle, bone, neurotrophin

## Abstract

Oxytocin (Oxt), osteocalcin (Ost), and NGF/BDNF have a role in bone homeostasis, reproduction, and cognition. Oxt/Ost is required for muscle repair. We investigated gene response of muscle and the inter-organ communication following cold stress (CS). The mRNA quantity of *Ngf*, *Ost*, *Oxt*, *Bdnf*, *p75ntr*, *Ntrk1*, *Gprc6a*, *Oxtr*, *Ntrk2*, *UCP1*, and *Il-6* genes in bone, brain, soleus (SOL), and *tibialis anterior* (TA) muscles from adult mice following CS were investigated. The myosin heavy-chain *Mhc2b*, *Mhc1*, *Mhc2x*, and *Mhc2a* gene expression were investigated. Mice were maintained at *T* = 23°C or 4°C for 6 h and 5-days (5d). CS mice did not show signs of muscle degeneration. An upregulation of *Ucp1* and *Ngf* genes by 2 and 1.5 folds, respectively, in TA after 6 h CS and *Ntrk1* by 4 and 22 folds in SOL muscle after 6 h and 5d CS, respectively, was observed; while after 6 h CS *p75Ntr* was downregulated in either muscle. *Bdnf* was unaffected, while after 5d CS *Ntrk2* was upregulated in TA. *Ost* was downregulated in SOL by 0.9-folds at 5d. Following 5d CS, *Oxtr* and *Il-6* genes were upregulated, respectively, by 1 and 1.5 folds in SOL. A downregulation of *Mhc2b*, respectively, by 0.96 and 0.88-folds after 6 h and 5d CS in SOL and *Mhc2a* was also downregulated by 0.88-fold after 5d CS in TA. *Mhc1* and *Mhc2x* were not affected. Changes in the expression levels of genes in TA and SOL muscles, bone, and brain following CS were regulated by IL6 and Oxt. CS potentiates the slow-twitch phenotype of SOL which is in line with the metabolic need of this muscle, and the potentiation of the slow-twitch phenotype in TA. Oxt and IL6 coordinate a phenotype-dependent tonic effect of slow-twitch muscle and Oxt regulates the inter-organ interaction between brain and SOL muscle. Muscle tropism is maintained by NGF signaling following CS.

Oxytocin, the neurotrophines NGF/BDNF and osteocalcin (Ost) are equally implied in regulating the physiological adaptation of the organism to challenging stimuli (Karsenty, 2012a; Hristova, 2013; Elabd et al., 2014; Mera et al., 2016a; Camerino et al., 2018). In physiological conditions, the expression levels of osteocalcin, NGF/BDNF, and their relative receptors Gprc6a, NGFR, NTRK1, and NTRK2 are linearly correlated in brain, testis, and BAT of both males and female mice supporting the idea that an inter-relationship between tissues could exist (Camerino et al., 2016). A link between BDNF and osteocalcin pathways has already been suggested to such an extent that BDNF is actually considered also as an osteocalcin target gene (Khrimian et al., 2017a,b). For instance, *Bdnf*^2lox/2lox/93^ mice selectively lacking BDNF gene in brain show not only central nervous system effects like anxiety but also high bone mass and hyperphagic obesity not related to adrenalin or serotonin (Ducy et al., 2000; Camerino et al., 2012). As well, osteocalcin is shown to be necessary for hippocampal memory and to prevent anxiety.

We recently demonstrated that cold-stress (CS) challenge can induce coordinated changes in the mRNA levels of *Ngf/p75ntr-Ntrk1*, *Bdnf-Ntrk2*, osteocalcin-*Bglap*/*Gprc6a*, and oxytocin-*Oxt/Oxtr* genes in bone, brain, testis, and BAT in adult mice (Camerino et al., 2018). In particular, using an animal model of thermogenic insult, we found that *Ucp-1* gene potentiation in BAT is associated with *Ngf* upregulation and trophic action in bone and testis. Instead, the related receptor gene *Ngfr (p75ntr)* was found to mediate an adaptation to CS through a feed-back loop in BAT. Moreover, it was shown that BDNF has bone and neuroprotective effects in this condition whereas higher expression of BDNF was observed in paraventricular nuclei (PVN) (An et al., 2015). As NGF, also osteocalcin and oxytocin were shown to exert a beneficial effect on bone and brain after a thermogenic insult. Oxytocin receptor is reported to mediate thermoregulation through a feed forward loop following CS in brain (Harshawa et al., 2017; Camerino et al., 2018). The observed coordinated changes of the mRNA levels of different genes may offer the advantage that lack of actions of a gene can be compensate by other genes with similar functions (Karsenty and Oury, 2012b; Camerino et al., 2018).

These thermogenic challenges have some similarities with those observed after a prolonged aerobic muscle exercise. In both cases, different organs became involved, modifying whole homeostasis. Consequently, the inter-organ communication is a critical mechanism to orchestrate such a complex process. Identifying molecules that could mediate the organ crosstalk during exercise or thermogenic challenge is a task of importance to understand the regulation of energy metabolism (Karsenty and Mera, 2017). Among these, a relationship between skeletal muscle and bone can be hypothesized considering that bone-derived hormones are also muscle regulators (Karsenty and Mera, 2017). In example, oxytocin is secreted by bone cells and bone cells express oxytocin receptor (Elabd et al., 2008); nevertheless, it is also required for muscle regeneration. Downregulation of oxytocin in young animals reduces muscle regeneration while administration of oxytocin improves this process, enhancing aged muscle stem cell activation and proliferation. Furthermore, an age dependent decline in oxytocin levels is observed despite the presence of normal oxytocin receptor activity that remains in the old tissues promoting myogenesis (Elabd et al., 2014). Consistent with this finding, oxytocin deficient mice show obesity and low sympathetic tone however in the absence of hyperphagia, a condition that can resembles aging. Conversely, the exogenous administration of oxytocin may augment the physiological function of the body (Camerino, 2009a). As regard, osteocalcin maintain muscle mass in older mice (Mera et al., 2016b) and promote protein synthesis in mouse myotubes through the activation of mTOR and AKT (Mera et al., 2016b). Interestingly, the undercarboxylated osteocalcin enhances during aerobic exercise whereas insulin level decreases. Osteocalcin signal in myofibers accounts for most of the exercise-induced release of interleukine-6, a myokine involved in the adaptation to exercise (Mera et al., 2016b). These notions lead to the hypothesis of the existence of a feed forward loop between bone and muscle promoting adaptation to exercise *via* osteocalcin and IL-6, respectively (Karsenty and Mera, 2017). Nevertheless, whether the actions of osteocalcin in skeletal muscle are mediated by osteocalcin receptor or by other related signaling pathways previously seen in brain (Khrimian et al., 2017a,b), is actually not known.

In rodent, four myosin heavy chain (MyHC) isoforms have been identified in skeletal muscle such as MyHC1, 2A, 2X, and 2B (Pette and Staron, 2000). MyHC1 is expressed in type 1, or slow-type, muscle fibers and composes the soleus muscle. Types MyHC2A, 2X, and 2B compose the fast-twitch *Extensor digitorum longus* or *tibialis anterior* (TA) muscles (Zierath and Hawley, 2004). Fibers expressing MyHC2A and 2X show intermediate properties between type 1 and type 2B. The MyHC2X fibers are fast-twitch glycolytic fibers, and type 2B fibers show a more marked fast-twitch and glycolytic phenotype than type 2X (Termin et al., 1989; Rivero and Talmadge, 1998; Rivero et al., 1999). Cold exposure induces an increase in energy expenditure and fatty acid catabolism in animals (Paul and Holmes, 1973; von Praun et al., 2001; Synak et al., 2011). A severe cold stress-full condition following a 4-week exposure at 4°C increased the slow-type MyHC1 content in the slow-type fiber of soleus muscle, while the intermediate-type MyHC2A content is reported to be reduced up-regulating the mitochondrial gene regulators in rat. Atrophy can be also observed (Mizunoya et al., 2014).

Thus, cold exposure and prolonged aerobic exercise share similar metabolic need increasing oxidative metabolism and lipolysis. As consequence, we hypothesized that cold exposure could potentiate slow fiber type as observed with prolonged exercise, and that oxytocin/osteocalcin/IL-6 or NGF/BDNF may regulate this process following CS. Actually, the role of Ost/Gprc6a, NGF/NGFR/NTRK1, BDNF/NTRK2, and Oxt/Oxtr in regulating the response of different muscle phenotypes to thermogenic challenge are not known. Moreover, in a stretch of mind and since Oxy function as utero-tonic is well-known (Arrowsmith et al., 2012), we generated the hypothesis that oxytocin tonic effect could extend also on other slow-twitch muscles as the soleus.

In this study, we investigated the relationship between Ost/Oxt and NGF/BDNF genes in regulating the response of different muscle phenotypes to thermogenic challenge. The mRNA levels of *Ngf*, *Bglap (Ost)*, *Oxt*, *Bdnf* and their receptors (*p75ntr*, *Ntrk1*, *Gprc6a*, *Oxtr*, *Ntrk2*), *Ucp1* and *Il-6* in bone, brain, soleus (SOL) and *tibialis anterior* (TA) muscles and the correlation curve between these organs from adult mice (3 months-old) exposed to 4°C, for short term (6 h) and long term (5 days) cold stress (CS) were investigated. The myosin heavy chain *Myhc2b* (fast-glycolytic), *Myhc1* (slow-oxidative), *Myhc2x* and *Myhc2a* (fast-glycolytic-oxidative) expression were also investigated. Linear correlation analysis between the changes of mRNA levels of gene of control and CS treated mice were performed in the absence/presence of the data of candidate gene in order to evaluate the contribution of each gene to the observed relationships.

## Results

### Gene Expression in Cold Stress Mice

**Figure 1:**
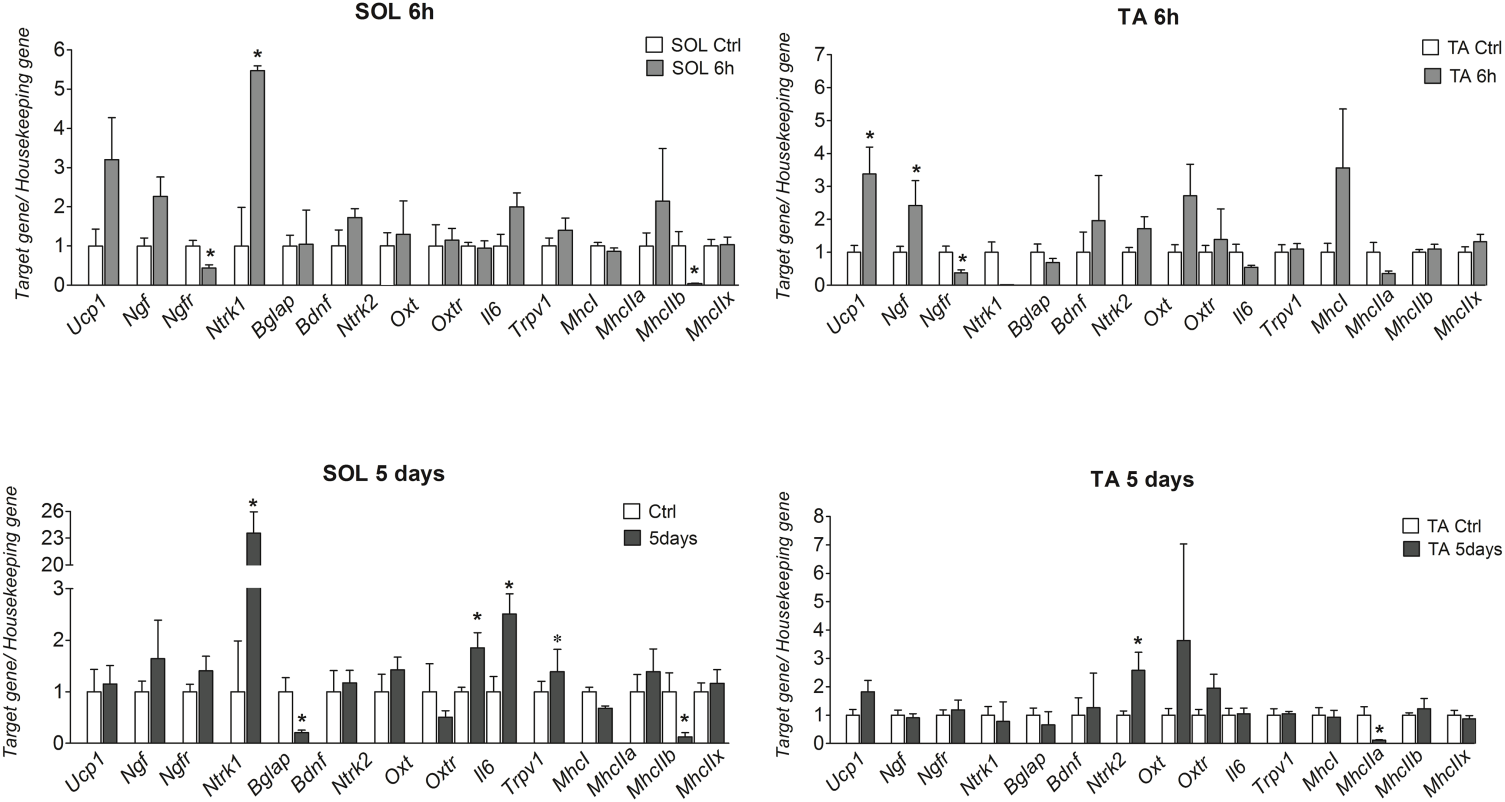
mRNA levels of *Ucp-1*, *Ngf*, *Ngfr (p75ntr)*, *Ntrk1*, *Ntrk2*, *MhcI/IIa-b-x*, *Bdnf*, *Oxt*, *Oxtr*, *Bglap (Ost)*, and *Gprc6a* genes in SOL and TA muscles in mice after 6 h and 5 days of cold stress. The data were expressed as mean ± SEM of a minimum of three and a maximum of five samples for each bar. ^*^Data significantly different vs. controls Student’s *t*-test (*p* < 0.05).

*Ucp1* and *Ngf* genes were significantly upregulated by 2 (*p* = 0.0162) and 1.5 (*p* = 0.0391) folds, respectively, in TA after 6 h CS. The mRNA levels of these genes were however not significantly affected following 5 days CS (Figure 1). The NGF gene receptor *Ntrk1* was significantly upregulated by 4 (*p* = 0.0462) and 22 (*p* = 0.0058) folds, respectively, in the SOL muscle after 6 h and 5 days CS vs. controls (Figure 1), while *p75Ntr* was down-regulated in both muscles after 6 h CS. In contrast, *Bdnf* gene was not affected in either muscle phenotypes but the *Ntrk2* gene was significantly upregulated following 5 days CS in TA muscle (Figure 1).

*Oxtr* and *Il-6* genes were upregulated, respectively, by 1 (*p* = 0.0176) and 1.5 (*p* = 0.0375) folds after 5 days CS in SOL but not in TA muscles vs. controls. *Bglap (Ost)* decreases significantly in SOL by 0.9-fold (*p* = 0.0198) after 5 days CS vs. controls, while osteocalcin *Gprc6a* gene receptor was not amplified in our samples, thereby suggesting that this gene was not expressed in TA and SOL in our experiments in contrast to other tissues (Camerino et al., 2018).

The fast-twitch glycolytic *Myhc2b* isoform was significantly down-regulated in SOL, respectively, by 0.96 (*p* = 0.0267) and 0.88 (*p* = 0.042)-folds after 6 h and 5 days CS vs. controls; *Myhc2a* was also significantly down-regulated by 0.88-fold (*p* = 0.0382) after 5 days CS in TA. However, *Myhc2x* was not affected following CS in SOL and TA muscles. The slow-twitch oxidative *Myhc1* isoform was also not significantly affected in either muscle phenotypes.

These findings suggest that 6 h and 5 days CS challenge specifically potentiates the slow-twitch phenotype increasing the ratios *Myhc1/Myhc2b* and *Myhc1/Myhc2a* in SOL and TA muscles, respectively.

In control mice, the *Myhc1/Myhc2b* ratio of SOL muscle was 3.37, and it was 250 and 70.5 after 6 h and 5 days CS. In TA muscle, the *Myhc1/Myhc2a* ratio was 0.012 in controls, and it was 0.12 after 6 h and 5 days CS. These data indicate that this state of energetic demand triggers the shifts of TA muscle toward the slow-twitch phenotype while potentiate the slow-twitch phenotype of SOL.

**Figure 2:**
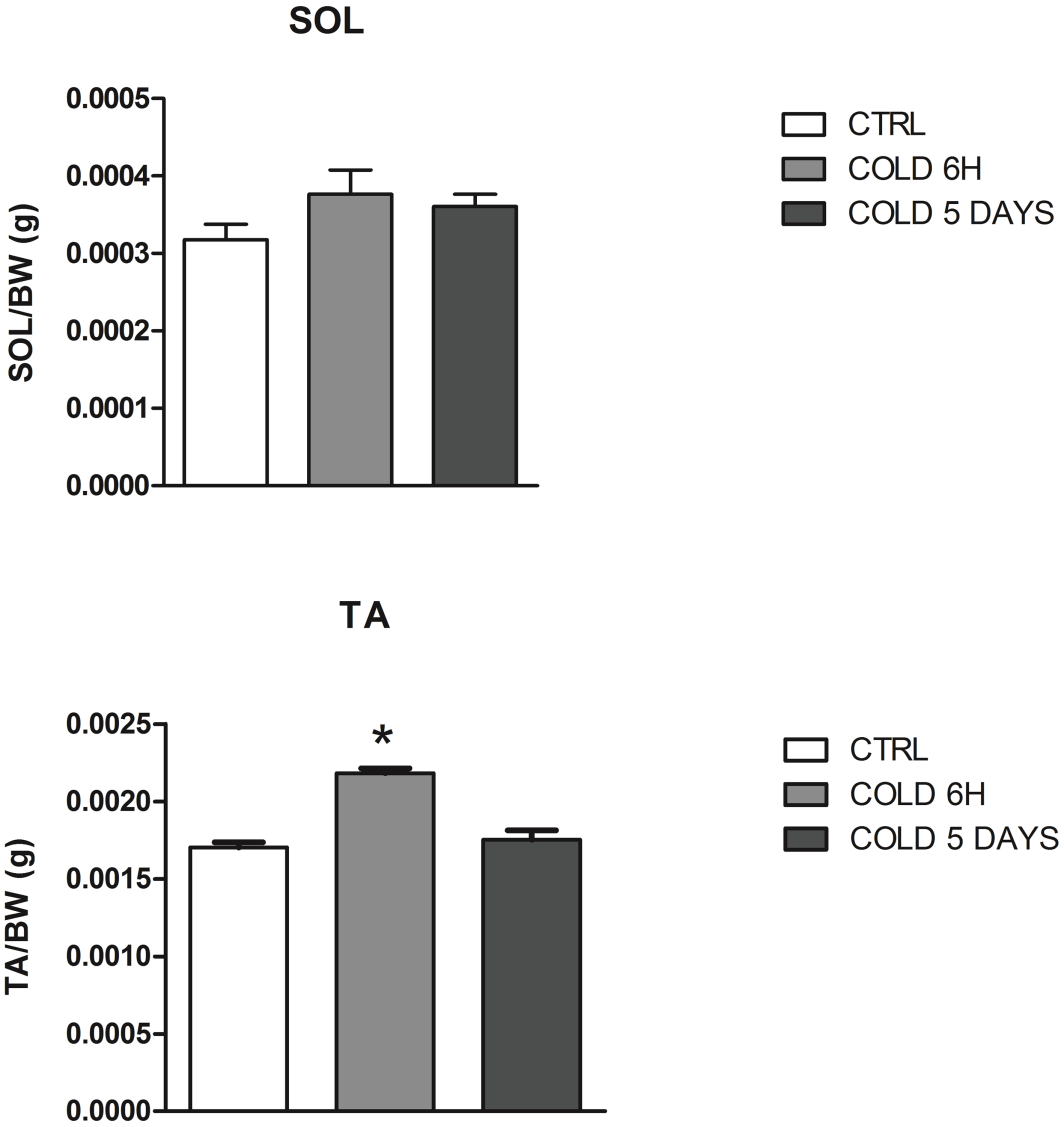
Muscle to body weight changes of SOL and TA muscles after 6 h and 5 days of cold stress in mice. The data were expressed as mean ± SEM of 5 mice. *Data significantly different vs. controls by Student’s *t*-test (*p* < 0.05).

The animals exposed to CS show a transient change in body weight, abdominal fat pad, and food consumption as previously shown (Camerino et al., 2018). The mean dried weight of SOL muscle was not affected by CS while the TA muscle weight significantly increased after 6 h CS (Table 1), suggesting that atrophy is not observed in these muscle phenotypes following 6 h and 5 days CS challenge (Figure 2). No effects were observed on the *Trpv1* gene mediating thermal and pain sensation in these muscles following CS challenges. These observations suggest that the muscle and tissues were preserved in this animal model of metabolic induced thermal stress.

**Table 1:** Change of muscle weight of mice following cold stress challenge.

Muscles	Controls	6 h of cold stress	5 days of cold stress
*Soleus* (SOL)	9.228 ± 3.792 (mg)	10.29 ± 2.457 (mg)	10.36 ± 1.415 (mg)
*Tibialis anterior* (TA)	50.58 ± 3.567 (mg)	59.50 ± 5.518^*^ (mg)	49.87 ± 5.411 (mg)

*Data significantly different vs. controls by Student’s *t*-test*.

* *p < 0.05*.

*Data reported as mean ± SEM*.

Regression analysis showed that changes in the expression levels of the genes of mice exposed to CS vs. control mice were linearly correlated showing a positive slope and coefficient of correlation close to 1 in TA muscle at 6 h and 5 days and in SOL muscle at 5 days.

In order to understand the relative contribution of a specific gene to this effect in SOL and TA muscles, we performed correlation analysis between the two variables computing the linear regression equation eliminating the expression data for each gene in the Excel electronic datasheet. No differences were found in either muscle phenotypes on correlation parameters computing the equations in the absence of *Bdnf*, *Oxt*, *Bglap (Ost)* genes and their receptors and of *Ngf* and *UCP1* genes after 6 h and 5 days CS. Similarly, no effects were observed in the calculated *R*^2^ of the equation following the elimination of the *Myhc1*, *Myhc2b*, *Myhc2x*, and *Myhc2a* mRNA data. While, upon the elimination of *Il6* expression data from the correlation electronic datasheet markedly decreased the *R*^2^ of the equation in SOL muscle after 6 h and 5 days CS. The *R*^2^ of the equation was mildly improved computing this parameter in the absence of *Il6* expression data in TA muscle after 5 days CS and not affected after 6 h CS (Table 2).

**Table 2:** Linear regression table of the change of gene expression of control mice vs. mice following cold stress challenge in skeletal muscle.

Muscles	6 h of cold stress	5 days of cold stress
Soleus (SOL) all gene data	*y* = 0.6734*x* + 0.3418 *R*^2^ = 0.594	*y* = 1.2275*x* − 0.05 *R*^2^ = 0.9338
SOL in the absence of Il6 gene data	*y* = 0.7995*x* + 0.3183 *R*^2^ = 0.1758	*y* = 0.8002*x* + 0.0293 *R*^2^ = 0.7405
*Tibialis anterior* (TA) all gene data	*y* = 0.6634*x* + 0.0281 *R*^2^ = 0.9654	*y* = 0.4369*x* + 0.0401 *R*^2^ = 0.8877
TA in the absence of Il6 gene data	*y* = 0.9087*x* − 0.0017 *R*^2^ = 0.9598	*y* = 0.7887*x* − 0.0027 *R*^2^ = 0.9758

These findings suggest that Il6 sustains the existing correlation in the SOL muscle being positively correlated with the neurotrophines, osteocalcin, and oxytocin genes in the slow-twitching muscle after 6 h and 5 days CS and mildly correlated with these genes in the fast-twitching phenotype after 5 days CS.

On the basis of the observed significant upregulation of the oxytocin receptor gene in SOL muscle, we hypothesized a possible involvement of the oxytocin signaling in the determination of the muscle phenotype following CS.

To evaluate the potential contribution of OXT to the determination of the slow-twitch phenotype in SOL muscle following CS and test the inter-organ communication hypothesis, we performed RT-PCR expression analysis in brain expressing high levels of *Oxt* gene and SOL muscle expressing high levels of *Oxtr* gene in the same plate of reaction to minimize factors enhancing co-variances affecting experimental data. Linear correlation was observed between gene data of these tissues after 6 h of CS, while a poor correlation was observed following 5 days CS showing a coefficient of correlation <0.5 (Table 3). The correlation between gene data of SOL and brain was lost computing the linear regression equation in the absence of the expression data of *Oxt* gene in brain after 6 h CS while computing the linear regression equation in the absence of the expression data of *Oxt* gene in brain after 5 days CS improved the correlation between gene data of SOL and brain (Table 3). No effects were observed computing the equation in the absence of all other gene data in brain and SOL muscle.

**Table 3:** Linear regression table of the gene expression of mice following cold stress challenge in brain and soleus muscle and bone and *tibialis anterior* muscle.

Genes	Brain after 6 h cold stress	Soleus muscle after 6 h cold stress	Brain after 5 days cold stress	Soleus muscle after 5 days cold stress
*Ucp1* *Ngf* *Ngfr* *Ntrk1* *Ost* *Gprc6a* *Bdnf* *Oxt* *Oxtr* *Il6* All gene data In the absence of OXT brain and muscle gene data	0.008315 1.213 0.03678 0.006 0.083 0.0009747 8.76501 13.895 0.01999 2.5431 *y* = 0.1769*x* + 0.3609 *R*^2^ = 0.6366 *y* = 0.1034*x* + 0.4206 *R*^2^ = 0.1843	0.991 0.987 0.001231 0.000001 0.2345 0.0003217 0.89111 3.213 0.000218 1.9912	0.0089074 0.9234 0.03541 0.00501 0.07301 0.00010749 2.1342 5.190121 0.020112 2.60094 *y* = 0.0996*x* + 0.5327 *R*^2^ = 0.0456 *y* = 0.6444*x* + 0.2926 *R*^2^ = 0.6594	1.4561 1.213 0.00144055 0.00001 0.195403 0.00036275 1.543001 0.007 0.00349289 2.01006
**Genes**	**Bone after 6 h cold stress**	***Tibialis anterior* after 6 h cold stress**	**Bone after 5 days cold stress**	***Tibialis anterior* after 5 days cold stress**
*Ucp1* *Ngf* *Ngfr* *Ntrk1* *Ost* *Gprc6a* *Bdnf* *Oxt* *Oxtr* *Il6* All gene data In the absence of *Il6* brain and muscle gene data	0.00079074 1.2123234 0.03541 0.00501 4.5671 3.32101 4.9301 0.5321 0.020112 5.19001 *y* = 2.6143*x* + 1.1905 *R*^2^ = 0.3758 *y* = 3.017*x* + 0.6342 *R*^2^ = 0.652	0.2165494 0.1223234 0.0144055 0.00001 0.9871 0.00032215 1.54021 0.12301 0.0349289 0.07101	0.000654 1.1987 0.0367 0.00501 4.5671 3.3421 4.9312 0.5321 0.020112 5.18794 *y* = 0.1233*x* + 0.1549 *R*^2^ = 0.2612 *y* = 0.199*x* + 0.1127 *R*^2^ = 0.5354	0.2685494 0.9121 0.01874055 0.0001 0.965471 0.00022115 1.5411 0.1871 0.0328289 0.06571

Therefore, brain *Oxt* sustains the existing correlation between gene data of SOL and brain at 6 h CS as demonstrated by the loss of correlation upon elimination of brain *Oxt* gene data. *Oxt* is however responsible for the poor correlation observed after 5 days CS as demonstrated by the improvement of *R*^2^ factor following elimination of brain *Oxt* gene data. These findings suggest that brain OXT may up-regulate the short-term response to SOL at 6 h, while may down-regulate the brain-SOL inter-communication following 5 days CS. Low circulating OXT levels are expected for a better response to 5 days CS challenge. Despite this, this effect is balanced by the significant up-regulation of the *Oxtr* gene found in the SOL muscle at 5 days in our experiments to maintain the OXT signaling. The increase of mRNA level of *Oxtr* at 5 days compounds the reduced level of circulating OXT consistent with previous studies (Elabd et al., 2014). These findings suggest that brain OXT mediates the biphasic “Oxytonic” signaling in the slow-twitch muscle following CS challenge.

To evaluate the contribution of *Oxt/Bglap(Ost)/Ngf/Bdnf* to the determination of the fast-twitch phenotype in TA muscle following CS, we performed RT-PCR expression analysis in bone and TA muscle in the same plate of reaction to minimize co-variances affecting experimental data. No correlation was however observed between gene data of these tissues after 6 h and 5 days CS (Table 3). The correlation between gene data of bone and TA was largely improved computing the *R*^2^ values in the absence of the *IL6* muscle data from the datasheet. No effects were observed computing the equation in the absence of all other gene data in these tissues. These findings suggest that *IL6* gene is responsible for the lack of correlation between genes in bone and TA muscle suggesting that circulating IL6 released from skeletal muscle may regulate the expression of *Oxt/Bglap(Ost)/Ngf/Bdnf* genes and the bone TA inter-organ communication through a feedback loop between these organs.

The significant upregulation of the *Ntrk2* gene found in TA muscle following 5 days CS suggests that BDNF may participate in determining muscle phenotype. Therefore, brain *Oxt* sustains the existing correlation between gene data of SOL and brain at 6 h CS.

## Discussion

In this study, we show that skeletal muscle properties are influenced by genes that are not classically associated with it and that skeletal muscle cross-talk with bone and brain allows adaptation of these tissues during thermogenic challenge (Monod and Jacob, 1961; Abboud, 2010). *Ngf* and *Ucp1* genes are upregulated in skeletal muscles. In particular, in slow-twitching muscle, the marked up-regulation of the neurothrophin receptor gene *Ntrk1* may lead to a sustained NGF signaling in response to circulating NGF released from bone and fast-twitching muscle during CS challenges; thereby skeletal muscle may be considered as a recipient of hormonal inputs released from bone under CS (Camerino, 2009a).

A novel finding is that oxytocin and its receptor, which are expressed in bone (Petersson et al., 2002) exert phenotype-dependent effects toward slow-twitch muscle. In SOL, indeed, it was observed an up-regulation of OXTR leading to oxytonic action following CS challenge. Six hours and 5 days CS induces down-regulation of *Myhc2b* in the slow-twitch SOL muscle which is in line with the metabolic urge of the slow-twitch oxidative muscle following thermogenic challenge. In addition, we show that *Oxtr* highly expressed in slow-twitch muscle is enhanced during 5 days CS. These effects can be mediated by feed-forward regulation of brain released oxytocin in SOL muscle. These observations acquire a paramount importance considering that the role of oxytocin in tissue homeostasis and regeneration is poorly documented. Previously, oxytocin up-regulation in skeletal muscle has already been observed following androgen treatment (De Jager et al., 2011), and it has been proposed as a mediator of muscle regeneration and maintenance in sarcopenia (Camerino, 2009b; Elabd et al., 2014). Therefore, we propose that oxytocin may exert tonic action on slow-twitch muscle similarly to what occurring in uterus (Arrowsmith et al., 2012). Slow-twitch fibers are indeed more resistant to mechanical and metabolic insults as compared to the fast-twitching fibers, so that this oxytonic action on SOL muscle promoted by oxytocin may lead to a more resistant phenotype against CS challenge. We also propose this oxytocin circuit being mediated by the homeostatic relation between brain and SOL through a time dependent feed-forward/feed-back regulation between these organs. This concept emerges from the loss of correlation between gene data of SOL and brain upon elimination of oxytocin gene in brain after 6 h CS, and the gain of correlation between these organs upon elimination of oxytocin in brain after 5 days of challenge.

We found that long-term cold stress for 5 days induced also a down-regulation of *Myhc2a* of the TA muscle living unaltered the slow-twitch *Myhc1*. This can be mediated by IL6 for instance; IL6 plays a significant contribution on muscle adaptation to CS as shown by the evidence that upon elimination of *IL6* gene data in SOL, the correlation between genes is decreased at both time points, while it is ameliorated in TA at 6 h. The feed-forward regulation involving IL6, muscle, and bone is consistent with previous studies (Mera et al., 2016b), although we failed to detect *Gprc6a* mRNA in SOL and TA in our work by RT-PCR. Interestingly, the *Ntrk2* gene significantly increased in TA at 5d CS. In this regard, we found a correlation between *Bglap(Ost)* and *Bdnf* genes in brain in control mice and we hypothesized that osteocalcin may use the BDNF receptor to exert its action in those tissues lacking osteocalcin receptor (Camerino et al., 2016). The interaction between *Bglap(Ost)* and *Bdnf* genes is supported by several studies (Khrimian et al., 2017a,b). In our experiments, the Ntrk2 in TA muscle may receive the BDNF and/or osteocalcin signaling released from bone and brain. Low osteocalcin serum levels in human is associated with an increased risk of diabetes type 2 as osteocalcin increases insulin sensitivity in human and animals, and insulin sensitivity is improved after cold stress (Chevalier et al., 2015; Mera et al., 2016b; Confavreux, 2018). In our experiment, *Bglap(Ost)* mRNA increase in bone may cause increased insulin sensitivity observed after cold stress in previous studies (Confavreux, 2018). Osteocalcin expression increases in bone following cold stress and triggers the expression and secretion of IL-6 in skeletal muscle, which in turn increases the production of bioactive osteocalcin in a feed-forward loop between bone and muscle (Karsenty and Mera, 2017).

We failed to show a significant correlation between gene data of bone and TA. Interestingly, we observed that the correlation between gene data of bone and TA is gained computing the *R*^2^ values in the absence of *IL6* muscle gene data. These findings suggest that *IL6* gene is responsible for the lack of correlation between genes in bone and TA muscle suggesting that circulating IL6 released from skeletal muscle may damper this inter-organ communication. The circulating IL6 may be a buffering mechanism regulating the bone-TA muscle signaling through feed-back regulation.

In conclusion, in this study, it emerges that brain oxytocin can mediate tonic effects on slow-twitch muscle through upregulation of its receptor, and that bone and muscle interaction is regulated by IL-6 in TA. NGF may play trophic and protective roles in muscle as well as BDNF in the fast-twitching muscle (Taheri Chadorneshin et al., 2017).

At the light of this study, since oxytocin at physiological concentrations does not cross the Blood Brain Barrier (Camerino, 2009b), we propose that exogenous administration of oxytocin could enhance several physiological functions and supplement states of increased energetic physiological needs.

## Materials and Methods

### Animals Care and Cold Exposure

The Replacement, Reduction and Refinement principles of the 2010/63/EU law on Animal Protection Used for Scientific Experiments were applied in the design of the protocols. The experimental protocol was approved by a named institutional and/or licensing committee (Organization for Animal Health O.P.B.A.) which is the competent Authority of the University of Bari for animal health at 21 January 2019.

Adult male 3 month-old C57BL/6N mice were housed in conventional cages with a 12:12-h light/dark cycle with free access to water and standard diet. After 1 week of acclimation, mice (*n* = 15) were divided into controls group maintained at temperature of 23°C, exposed to cold (CS) at *T* = 4°C for 6 h and 5-days under the same diet and light conditions. Body weight and food intake in both controls and cold groups were daily measured. The animals were anesthetized *via* inhalation (induction with ~3% isoflurane and 1.5% O_2_ L/min) and sacrificed by cervical dislocation. Subsequently, from each animal the following organs where quickly isolated and weighed: whole brain, *tibialis anterior* (TA), soleus (SOL) muscles, and bone (femur). All organs were frozen in liquid nitrogen and stored at −80°C for RNA extraction.

### Real-Time PCR Experiment

The RNA extraction protocol and analysis chosen based on the amount and type of tissue as previously described (Tricarico et al., 2005, 2008, 2010; Mele et al., 2012). Briefly, the brain, bones (femur), and tibialis muscles RNA were extracted with Trizol (Invitrogen); the soleus muscle extraction was performed with RNeasy Tissue Micro Kit (Qiagen) (Tricarico et al., 2010). Real-time PCR was performed using the Applied Biosystems Real-time PCR 7500 Fast system (Camerino et al., 2016). The mRNA expression of the genes was normalized to the best housekeeping gene *Gapdh* selected within *Eef2*, *Hprt1*, *2 beta-microglobulin*, *Gapdh*, and *Actinb* by BestKeeper version. For low expressed genes, *Ngf*, *Bdnf*, *Ntrk1*, *Ntrk2*, *Ngfr*, *Bglap*, *Oxt*, *Oxtr*, *Gprc6a*, *Trpv1*, the pre-amplification protocol was performed by using TaqMan PreAmp MasterMix before the real time PCR experiments. TaqMan hydrolysis primer and probe gene expression were designed and synthesized from Thermo fisher scientific and they are described in Table 4 with the exception for *Actinb* (primer For: 5′-CCAGATCATGTTTGAGACCTTCAA-3, primer Rev: 5′-CA TACAGGGACAGCACAGCCT-3, probe: VIC-ACC CCA GCC ATG TAC GTA-MGB, the sequence target was NM_007393.5, with a amplicon length of 71 pb and an assay location in position 469). The RT-PCR protocols were performed in line with the guidelines for qPCR (Bustin et al., 2009).

**Table 4:** Gene probes for RT-PCR experiments.

Gene name	Gene symbol	Assay ID of Therm. Fisher. Scient.
Uncoupling protein 1 (mitochondrial, proton carrier)	*Ucp-1*	Mm01244861_m1
Nerve growth factor	*Ngf*	Mm01192897_m1
Nerve growth factor receptor (TNFR superfamily, member 16)	*Ngfr*	Mm01309638_m1
Neurotrophic tyrosine kinase, receptor, type 1	*Ntrk1*	Mm01219406_m1
G protein-coupled receptor, family C, group 6, member A	*Gprc6a*	Mm01192897_m1
Bone gamma-carboxyglutamate protein 3	*Bglap3*	Mm01741771_g1
BRAIN derived neurotrophic factor	*Bdnf*	Mm 04230607_s1
Neurotrophic tyrosine kinase, receptor, type 2	*Ntrk2*	Mm00435422_m1
Oxytocin	*Oxt*	Mm00726655_s1
Oxytocin receptor	*Oxtr*	Mm01182684_m1
Transient Receptor Potential Cation Channel Subfamily V Member 1	*Trpv1*	Mm01246300_m1
Eukaryotic translation elongation factor 2	*Eef2*	Mm 01171434_g1
Hypoxanthine guanine phosphoribosyl transferase	*Hprt-1*	Mm00446968_m1
Beta-2 microglobulin	*B2m*	Mm00437762_m1
Glyceraldehyde-3-phosphate dehydrogenase	*Gapdh*	Mm99999915_g1
Myosin, heavy polypeptide 7	*Myh7(Mhc1)*	Mm00600555_m1
Myosin, heavy polypeptide 2	*Myh2(Mhc2a)*	Mm00454982_m1
Myosin, heavy polypeptide 1	*Myh1(Mhc2x)*	Mm01332489_m1
Myosin, heavy polypeptide 4	*Myh4(Mhc2b)*	Mm01332541_m1

### Statistics

Significance between groups was evaluated by Student’s *t*-test (*p* < 0.05) using GraphPad Prism (v. 5). The gene expression data in control mice vs. mice following cold stress challenge were plotted. The linear regression equation *y* = *mx* + *b* was used, and the Pearson’s correlation coefficient was also calculated using Excel Software (Microsoft) electronic datasheet. The regression analysis was performed in the presence of all gene expression data. The calculation of the correlation coefficient (*R*^2^) was computed in the absence of the expression data for each gene to evaluate the contribution of a specific gene to the existing correlation between variables. Linear correlation analysis between biological variables has been successfully used to evaluate the contribution of gene and factors affecting specific functions in tissues and cells (Camerino et al., 2016, 2018).

## Data Availability Statement

The raw data supporting the conclusions of this manuscript will be made available by the authors, without undue reservation, to any qualified researcher.

## Ethics Statement

The animal study was reviewed and approved by Organization for Animal Health O.P.B.A. which is the competent Authority of the University of Bari for animal health at 21 January 2019.

## Author Contributions

CC designed the experiments. EC and AF performed the experiments. CC and DT analyzed the data and wrote the paper. MC, ML, and DT contributed reagents, materials, and analysis.

### Conflict of Interest

The authors declare that the research was conducted in the absence of any commercial or financial relationships that could be construed as a potential conflict of interest.
